# Fate and Prediction of Phenolic Secoiridoid Compounds throughout the Different Stages of the Virgin Olive Oil Making Process

**DOI:** 10.3390/antiox6030061

**Published:** 2017-08-03

**Authors:** Giuseppe Fregapane, M. Desamparados Salvador

**Affiliations:** Food Technology Department, Faculty of Chemistry, University of Castilla-La Mancha, E-13071 Ciudad Real, Spain; Amparo.Salvador@uclm.es

**Keywords:** phenolic secoiridoids, prediction, crushing, malaxation, olive paste, virgin olive oil

## Abstract

The evolution of the main phenolic secoiridoid compounds throughout the different stages of the virgin olive oil making process—crushing, malaxation and liquid-solid separation—is studied here, with the goal of making possible the prediction of the partition and transformation that take place in the different steps of the process. The concentration of hydroxytyrosol secoiridoids produced under the different crushing conditions studied are reasonably proportional to the intensity of the milling stage, and strongly depend on the olive variety processed. During malaxation, the content of the main phenolic secoiridoids is reduced, especially in the case of the hydroxytyrosol derivatives, in which a variety-dependent behaviour is observed. The prediction of the concentration of phenolic secoiridoids finally transferred from the kneaded paste to the virgin olive oil is also feasible, and depends on the phenolic content and amount of water in the olive paste. The determination of the phenolic compounds in the olive fruit, olive paste and olive oil has been carried out by LC-MS (Liquid-Chromatography Mass-Spectrometry). This improved knowledge could help in the use of more adequate processing conditions for the production of virgin olive oil with desired properties; for example, higher or lower phenolic content, as the amount of these minor components is directly related to its sensory, antioxidant and healthy properties.

## 1. Introduction

The extraction of virgin olive oil (VOO) is a critical process, as its operating conditions greatly affect the quality of the final product; moreover, it is not just a physical process that breaks the fruit’s tissues to free the oil droplets enclosed in the cell. Upon olive crushing, and during kneading, different enzymes involved in the generation and transformation of phenolics and volatile components are activated [1,2]. Therefore, the content of these minor compounds, closely related to the quality of VOO, can be modified when varying the processing conditions [3,4,5,6,7,8].

The appreciated sensory profile of this fruit juice, characterized by a unique aroma and taste (flavour), and noticeable nutritional and biological properties, are due to the content of its minor components, mainly volatile and phenolic compounds [8,9,10,11,12], which remain in the oil as only mechanical means for its extraction are used.

In fact, the fruity and green aroma of superior quality virgin olive oils is mainly produced by the volatile compounds generated by the lipoxygenase (LOX) pathway from polyunsaturated fatty acids [13]; whereas the phenolic components—also named polyphenols or biophenols—affect the taste, especially the positive bitter and pungent sensory attributes, as well as the oxidative stability and the nutritional and healthy properties of the VOO [14,15,16]. Indeed, a health claim for food labelling was approved a few years ago by the EFSA (European Food Safety Authority): “olive oil polyphenols contribute to the protection of blood lipids from oxidative stress” (European Community Regulation 432/2012) [17].

The two main families of complex phenolic compounds found in VOO (named secoiridoids) are the derivatives of hydroxytyrosol (the dialdehydic and aldehydic forms of elenolic acid linked to hydroxytyrosol, usually abbreviated as 3,4-DHPEA-EDA and 3,4-DHPEA-EA) and of tyrosol (the dialdehydic and aldehydic forms of elenolic acid linked to tyrosol, also known as p-HPEA-EDA and p-HPEA-EA; e.g., [18,19,20].

As is known, the olive variety—its basic genetics, together with other agronomical variables, which mainly consist of fruit ripening, geographical location, irrigation, soil and climate—determines the characteristics of the olive fruit, in terms of the content in precursors and enzymatic activities responsible for the generation and transformation of minor compounds; e.g., [21,22,23,24].

In recent years, several innovations and equipment prototypes have been investigated and proposed to improve the yield of the process or to produce changes in the composition and quality of the VOO produced. They are based on the use of inert gas or an oxygen control [25,26,27,28,29,30], heating assisted systems such as ultrasound, microwave or a pulsed electric field [31,32,33,34,35], or a better control of the olive paste temperature [36,37] during malaxation. Nevertheless, the oil industry is still using the continuous process, introduced no more than three to four decades ago, which employs a metal crusher, a malaxer and a horizontal centrifuge known as a “decanter”, which improved the productivity as compared to the previous pressing method thanks to the increased process capacity, which reduces the storage of the olives and therefore the fermentation processes, thus leading to superior quality oils [38]. Different types of crushers are available, such as hammer mills, toothed crushers or blade cutters; the hammer crusher is commonly identified as the strongest and yields bitter oils [4,39,40].

In the literature, only a few publications have analysed the relationship between the phenolic content in the olive fruit and the corresponding final VOO [7,23,41,42,43,44]. Some of them have studied the partition throughout the different stages of the process; however, none have approached prediction models for the phenolics, and this is therefore the first report that focuses on this challenge.

The goal of the experimental work carried out was therefore to analyse the feasibility of being able to predict the evolution of the content of the main families of polar phenolics in each stage of the VOO making process: crushing, kneading and solid-liquid separation by centrifugation. To this end, a batch of Cornicabra and Arbequina olives cultivars—known for their different minor component composition [23]—were processed under different conditions to obtain a set of different crushed olive pastes with different phenolic contents in order to be able to study their evolution during the further stages of the process. This research was performed at a laboratory scale for a better control of both processing conditions and sampling; however, to check and validate the approach and model proposed at an industrial scale, and employing several olive varieties and crop seasons, further investigation will be required.

## 2. Materials and Methods

### 2.1. Olive Fruit Sampling and VOO Processing

The research was performed using Arbequina and Cornicabra olives varieties cultivated in the centre of Spain (Ciudad Real area). Arbequina and Cornicabra olives were collected at a ripeness index of 2.8 and 4.5 respectively (since the Cornicabra cultivar is processed at a higher ripeness than Arbequina). Their oil content was 31.4% and 44.7% as dry weight, and the humidity was 50% and 35%, respectively.

800 g olive samples from batches of 40–50 kg were crushed using laboratory scale hammer mills at 1500 rpm and 3000 rpm, each equipped with fixed grids with different hole diameters (5, 6 and 7 mm). A blade cutter was also used.

The crushed pastes, obtained from the different techniques, were kneaded according to the Abencor procedure [45] under the same conditions of temperature (28 °C) and malaxation time (45 min). The kneaded pastes were then centrifuged at 3500 rpm and the oils recovered. Olive paste samples obtained were frozen at −70 °C until analysis. Olive oil samples were filtered with anhydrous Na_2_SO_4_ as a drying agent to preserve the samples from the oxidation, and stored at 4 °C in darkness using topaz glass bottles without head space prior to analysis.

### 2.2. Analytical Determinations in Olive Fruit and Paste

The water and fat content of the olive fruit in both cultivars was assessed according to the UNE Spanish Standard method (AENOR) [46].

The phenolic content of the fruit and olive paste was analysed in the following way: 4.0 ± 0.0001 g of sample was mixed with 4-hydroxyphenylacetic acid used as internal standard (2.0 ± 0.1 mg) in methanol/water (80:20 v/v) (40 mL) for 2 min with an Ultraturrax homogenizer (IKA-Werke GmbH & Co. KG, Staufen, Germany; 14,000 rpm). The suspension was shaken (20 min, 150 rpm, <4 °C in darkness), and then centrifuged (10 min, 4 °C and 3850× *g*). The hydromethanolic solution was recovered and filtered through a 0.45 μm nylon syringe filter. High-performance liquid chromatography (HPLC; Agilent Technologies 1100 series) equipped with an automatic injector, a column oven and a diode array UV detector was used to analyse the phenolic fraction. A ZORBAX SB-C18 column (250 × 4.6 id mm, 5 μm particle size) (Agilent Technologies, Santa Clara, CA, USA), maintained at 30 °C, was used with an injection volume of 20 μL and a flow rate of 1.0 mL/min. The mobile phase was of water/acetic acid (95:5 v/v) (solvent A) and methanol (Solvent B) with the following gradient: 95% A/5% B for 2 min, 75% A/25% B for 8 min, 60% A/40% B for 10 min, 50% A/50% B in 16 min, and 0% A/100% B for 14 min, for 10 min, and return to initial conditions for 13 min according to [25].

Phenolic compounds were identified by MS and UV-visible spectra and retention times of standard substances. A LCQ Deca XP Plus (*m*/*z* range: 50 to 2000 amu; Thermo Electron Corp., Waltham, MA, USA) mass detector equipped with an electrospray ionization system was used, with nitrogen as nebulizing gas at a flow rate of 14 units, and 250 °C and 4.50 kV as temperature and voltage of the capillary, respectively. Negative ionization mode was employed to acquire data and fragmentation was carried out using helium with a collision energy between 30% and 40%.

### 2.3. Analytical Determinations in Virgin Olive Oil

The quality indices—free acidity (oleic acid percentage), peroxide value (PV; meq O_2_/kg), and the K_232_ and K_270_ extinction coefficients at 232 and 270 nm—were determined by the methods described in the European Union standard methods and subsequent amendments (European Community Regulation 2568/91) [47].

For the phenolic compounds according to [18], 250 μL of 15 mg/kg of syringic acid in methanol, as internal standard, was added to 2.5 g of virgin olive oil and the solvent removed with a rotary evaporator at 35 °C under vacuum. The oil was then dissolved in 6 mL of hexane, and a diol-bonded phase cartridge (Supelco Co., Bellefonte, PA, USA) was employed to extract the phenolics. Methanol (6 mL) and hexane (6 mL) were used for the conditioning of the cartridge, and the oil solution was then applied. The SPE column was washed with hexane (2 × 3 mL) and with hexane/ethyl acetate (85:15, v/v; 4 mL); then, the phenols were eluted with methanol (15 mL) and the solvent was eliminated with a rotary evaporator at 35 °C under vacuum until dry. The phenolic residue was finally dissolved in methanol/water (1:1 v/v; 250 μL) and analysed by HPLC. A Chromolith performance RP-18 endcapped column (100 × 4.6 id mm) was used under the following conditions: 30 °C, 20 μL injection volume, and 2.0 mL/min flow rate. A mixture of water/acetic acid (95:5 v/v) (solvent A), methanol (B) and acetonitrile (C) was used as the mobile phase: from 2.5% (B)/2.5% (C) to 25% (B)/25% (C) in 25 min, to 100% (C) in 3 min maintained for 6 min, to 2.5% (B) and 2.5% (C) in 3 min at 1.2 mL/min flow and kept for 40 min at normal flow. Phenolic compounds were quantified at 280 nm using syringic acid as the internal standard; their response factors were established by Mateos el al. [18].

Regarding the origin of the standards, reagents and solvents, Oleuropein (>90% purity) was purchased from Extrasynthese (Genay, France); 4-hydroxyphenylacetic acid (98% purity), syringic acid (98% purity), and 4-methyl-2-pentanol (99%) were from Sigma-Aldrich (Steinheim, Germany). All the others common reagents were of the appropriate purity from various suppliers. HPLC grade methanol, acetonitrile and *n*-hexane were from Merk KgaA (Darmstadt, Germany). Ultra purity water was produced using a Millipore Milli-Q system.

All experiments and analytical determinations were carried out at least in duplicate.

## 3. Results and Discussion

**Figure 1:**
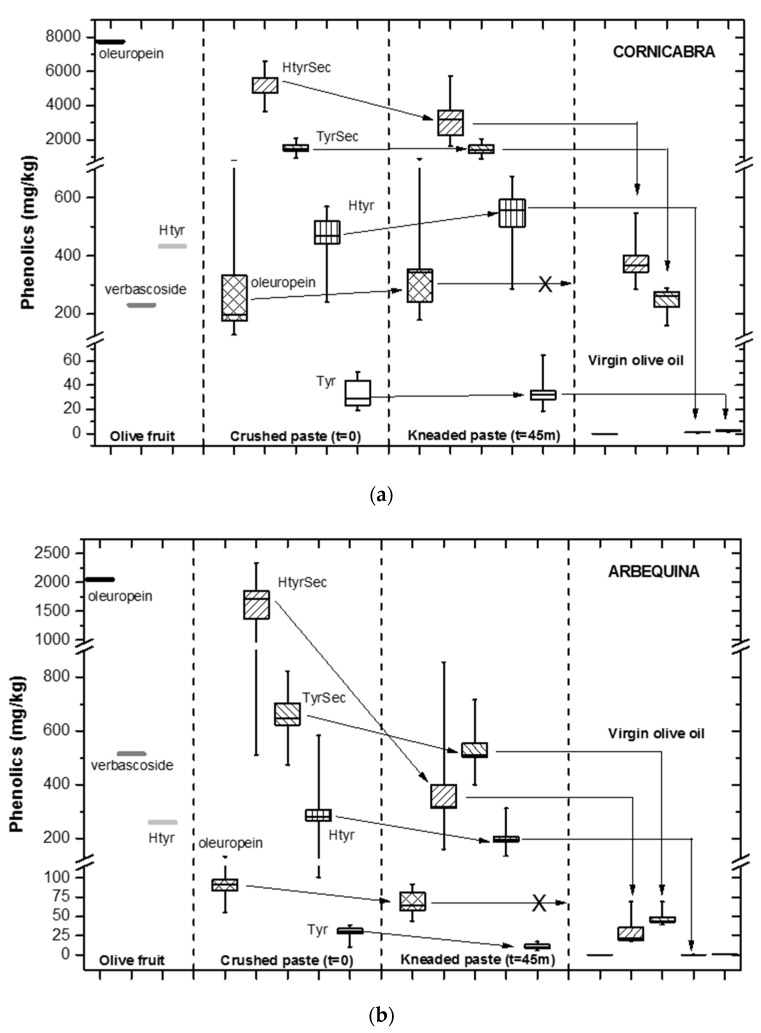
Overview of the fate of (**a**) Cornicabra virgin olive oil phenolics and (**b**) Arbequina virgin olive oil phenolics during the making process.

An overview of the fate of the major polar phenolics throughout the different stages of the virgin olive oil (VOO)-making process—the crushing of the olive fruit, the kneading of the olive paste (also known as malaxation), and finally the liquid-solid separation by centrifugation of the oily phase to yield a virgin olive oil directly ready for consumption—is depicted in Figure 1 for the two studied olive varieties, Cornicabra (Figure 1a) and Arbequina (Figure 1b).

As recalled in the introduction, the major polar phenolic compounds found in VOO are the complex form (called secoiridoids) of the hydroxytyrosol and tyrosol (abbreviated as HtyrSec and TyrSec, both in figures and text). These polar phenolics originate from the corresponding glucosidic forms—namely, oleuropein and demethyl-oleuropein [48]—which are present in very high concentration in the olive fruit (Figure 1). As known and depicted in Figure 1, the amount of these compounds in the final VOO is very low as compared to their content in the olive paste after crushing and during kneading. For instance, the ranges of concentration for HtyrSec found in this study in the VOO were 284–550 mg/kg and 18–69 mg/kg in Cornicabra and Arbequina oils, respectively, whereas in the crushed olive pastes a much higher content, ranging from 3669 to 6582 and 511–2334 mg/kg for each olive variety was measured; from ten to thirty times higher.

The two olive cultivars chosen for this research show great differences in their minor component profiles [23,49]: Cornicabra olives possess a much higher oleuropein content (7740 mg/kg) as compared to Arbequina (2050 mg/kg; as sum of oleuropein and its demethyl form; Figure 1).

As stated in the introduction, the goal of this work was to analyse the feasibility of being able to understand and predict the evolution of the content of the main families of VOO polar phenolics during the making process. To this end—as explained in material and methods—a batch of Cornicabra and Arbequina olives were processed using different intensities of crushing—like those used in the oil industry—with the purpose of producing a set of nine different olive pastes for each cultivar with a different olive paste phenolic content.

As expected, the quality indices of the virgin olive oils (VOO) produced in this research were far below the limits established by the European Commission Regulation 702/07 [50] for the extra virgin category (data not shown), as the fruits used were fresh, healthy and carefully processed.

### 3.1. Effect of Crushing

Crushing produces a profound transformation in the chemical composition of the phenolic compounds of the olive fruit (Figure 1), as described in literature by Artajo et al. (2007) and Inarejos-Garcia et al. (2011) [7,41]. This is caused by the activity of enzymes, such as β-glucosidase and esterase, related to the generation of phenolics [51].

Indeed, oleuropein decreased considerably; i.e., from 7740 down to 127–794 mg/kg in the case of Cornicabra (Figure 1a) depending on the intensity of milling. During crushing, oleuropein is transformed into its aglycone secoiridoid derivatives, which are contained both in the olive paste (i.e., HtyrSec 3669–6582 mg/kg and TyrSec 908–2064 mg/kg for Cornicabra; as depicted in the box plot of Figure 1a) and in the olive oil; on the other hand, these secoiridoid compounds are not detected in the olive fruit.

The stronger the conditions of crushing (i.e., higher rotation speed and smaller grid holes) the higher the concentration of phenolics in the olive paste in both varieties [7]. This may be explained by the better breakage of the fruit tissue and by the increased activity of enzymes related to the biogeneration of phenolic compounds such as β-glucosidase [52,53].

**Figure 2:**
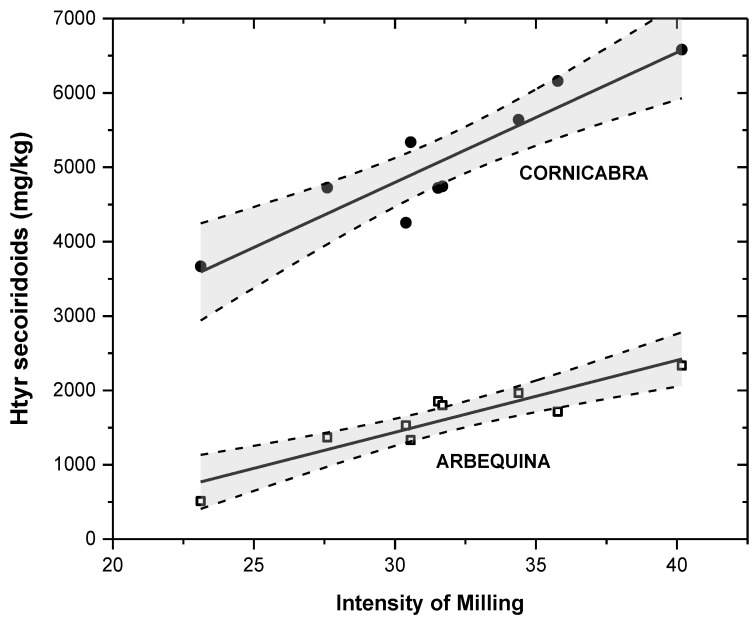
Effect of milling on formation of hydroxytyrosol secoiridoids in crushed olive paste.

If a scale establishing the intensity of milling is plotted—defined by the percentage yield of the transformation of oleuropein and demethyl-oleuropein into the tyrosol secoiridoids (since this family is more stable than that of hydroxytyrosol)—then it can be observed that the concentration of hydroxytyrosol secoiridoids produced under the different crushing conditions studied are reasonably linear for both olive varieties studied (r^2^ = 0.844, *p* < 0.01 for Cornicabra and r^2^ = 0.843, *p* < 0.01 for Arbequina), as depicted in Figure 2. This observation shows the feasibility and usefulness of such a type of scale—related to the intensity of milling—for its being able to reasonably predict the amount of secoiridoids yielded during the crushing operation (r^2^ = 0.833, *p* < 0.01 for Cornicabra and r^2^ = 0.887, *p* < 0.01 for Arbequina).

The regression lines for Cornicabra and Arbequina are not parallels (slopes of 175 and 97 respectively), meaning that the effect of crushing is not simply proportional to the initial content of phenolic precursors in the olive fruit, but strongly depends on the olive cultivar processed. This relevant difference in the transformation rate may be due to the different enzymes’ levels in each olive variety, which significantly affects the amount of secoiridoids produced during milling [30,54]. In fact, in a previous work of our research group [23], different ratios between the phenolic content for each olive fruit cultivar studied and the corresponding olive oil were observed; however, intermediate stages (crushing, malaxation and centrifugation) were not evaluated in that study.

### 3.2. Evolution during Malaxation

The kneading also produces an important effect on the concentration of phenolics in the olive paste, mainly due to the activity of oxidative enzymes such as polyphenol oxidase, peroxidase and lipooxygenase during malaxation [5,25,26]. After 45 min of kneading—the conditions employed, and commonly used by the industry—the content of the main phenolic secoiridoid components diminished, in particular for the hydroxytyrosol family; i.e., HytrSec from 3669–6582 down to 1630–5710 mg/kg for Cornicabra, or TyrSec from 473–823 to 400–717 mg/kg in Arbequina (Figure 1). This relevant difference between the two families is probably caused by their orthodiphenolic structure of HtyrSec, which is more sensitive to the enzymatic oxidation [25].

**Figure 3:**
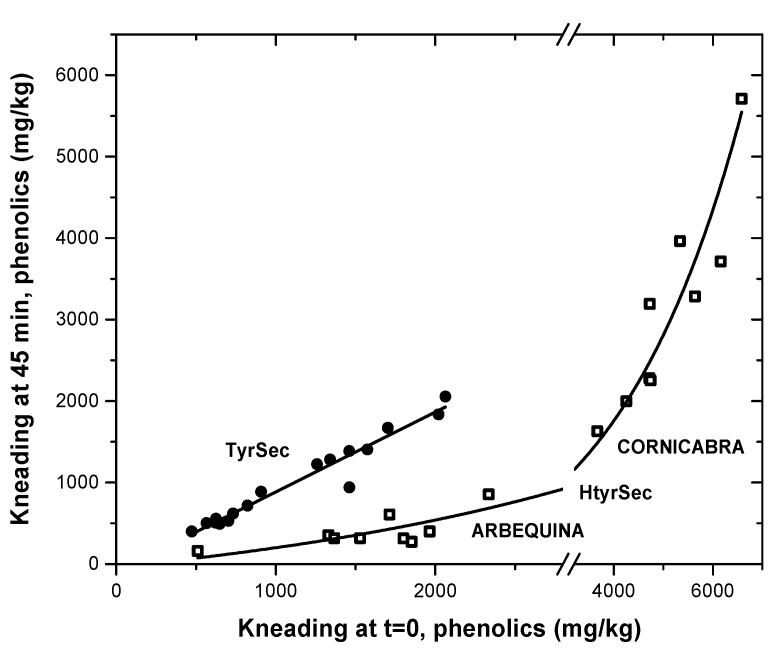
Effect of kneading on the evolution of hydroxytyrosol and tyrosol secoiridoids in the olive paste.

As depicted in Figure 3, the evolution of the Tyr secoiridoid concentrations, in all conditions and both olive varieties, followed a linear regression (r^2^ = 0,953; slope = 0.98; intercept, practically = 0), showing that a small reduction is observed when kneading; being, as known, more stable than HtyrSec. On the other hand, in the case of Htyr derivatives, an apparent exponential relationship is observed (r^2^ = 0.99; model 508^x + 0). However, if the values of each olive variety are analysed separately, a different behaviour emerges, and almost a linear relationship for each case is observed; with a slope of 1.20 for Cornicabra and 0.30 for Arbequina (Figure 3), in which once again, as in the case of crushing, the character of the olive cultivar assume a relevant role. The same behaviour was observed by Artajo et al. (2007) [41] in studying the evolution of the phenolic content in the Arbequina olive paste during a 15–45 min malaxation time.

### 3.3. Transfer to the Oily Phase

Finally, the partial solubility of the polar phenolic substances in the oil [53] drastically reduces their quantity in the final product (Figure 1). Thus, in Cornicabra, only between 284–550 mg/kg of HtyrSec and 159–288 mg/kg of TyrSec were measured in the virgin oil after the horizontal centrifuge (decanter) separation. Furthermore, oleuropein was not found in VOO since it is not soluble in the oily phase [53]. This trend agrees with other studies [7,23,41,42,43,44]. In the case of Arbequina, an average ratio of 14.0% between the phenolics in VOO and in the kneaded olive paste (45 min) and 4.4% between phenolics in VOO and in the crushed paste was obtained, very similar to that observed by Artajo et al. [41] for the same variety (12.4% and 3.2%). For Cornicabra, a mean ratio of 15.5% between phenolics in VOO and in kneaded olive paste and 8.7% between phenolics in VOO and in crushed paste, double that observed for Arbequina, was observed.

**Figure 4:**
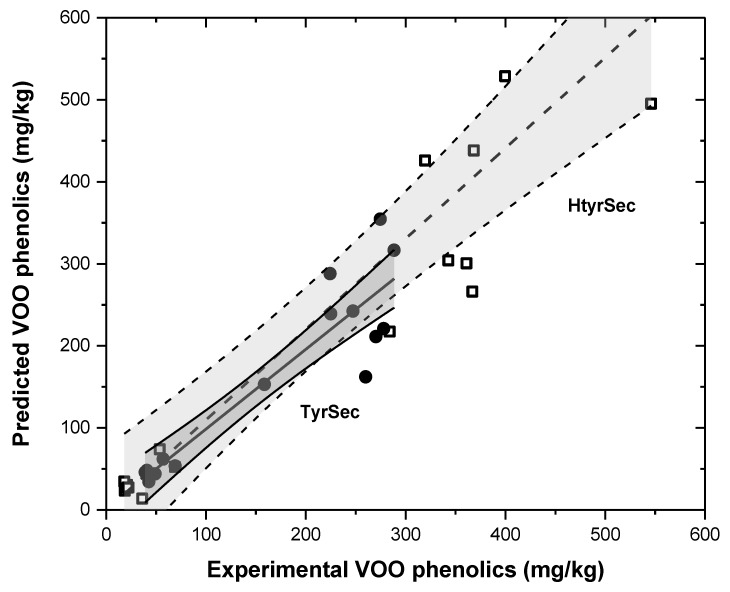
Prediction of hydroxytyrosol and tyrosol secoiridoids transfer to virgin olive oil.

If the relationship between the polar phenolic contents in the olive paste at the end of kneading and in the corresponding olive oil is analysed, an unclear relationship is observed (r^2^ = 0.70–0.75); moreover, a great difference is observed between the two olive varieties studied. However, if the partition of phenolics between the oily and water phases is taken into account, assuming that all the polar phenolic compounds are solubilized in the water phase present in the olive paste—constituted by the olive fruit humidity plus the water possibly added to the malaxer during processing—and that the content of these compounds in the oily phase depend only on their partition coefficient (phenolic conc. oil/phenolic conc. water [53]), a better linear regression for both secoiridoid families and olive varieties studied is observed and reported in Figure 4 (r^2^ = 0.810; *p* < 0.05; slope = 1.10; intercept practically = 0 for HytrSec; and r^2^ = 0.86; *p* < 0.05; slope = 0.97, intercept practically = 0 for Tyr secoiridoids). Average partition coefficients of 0.045 for total polar phenolics, 0.047 for HtyrSec and 0.064 for TyrSec, for the 18 olive pastes studied were observed.

This means that it is apparently feasible to predict the concentration of the phenolic secoiridoids that are transferred from the kneaded paste to the virgin olive oil, depending on the phenolic concentrations in the olive paste and the amount of water present: the humidity of the olive paste (35% and 50% for Cornicabra and Arbequina in this case) and the water possibly added during malaxation.

## 4. Conclusions

The present study shows that it is apparently feasible to predict the amount and type of phenolic compounds which evolve and are transferred along each of the different stages of the virgin olive oil making process: crushing, malaxation and liquid-solid separation. This improved knowledge could help in the use of adequate processing conditions for the production of VOO with desired properties—higher or lower phenolic content—as the amount of these minor components is directly related to the sensory, antioxidant and healthy properties of this highly appreciated product by consumers. This research has been carried out at a laboratory scale for a better control of both processing conditions and sampling; however, further investigation is required at an industrial scale, employing several olive varieties and crop seasons, in order to check and validate the approach and model proposed.
